# Supplementary material for: Meta-analysis reveals the predictable dynamic development of the gut microbiota in commercial pigs
Source: Microbiol Spectr. 2023 Oct 10;11(6):e01722-23. doi: 10.1128/spectrum.01722-23 (PMC10715009; doi:10.1128/spectrum.01722-23)
Supplement: Supplemental figure legends — Legends for Fig. S1 to S11. [file spectrum.01722-23-s0001.docx]

**Meta-analysis reveals the predictable dynamic development of the gut microbiota in commercial pigs**

Wenxuan Dong, Nicole Ricker, Devin B. Holman, Timothy A. Johnson

**Supplementary Figure Legends**

**FIG S1** Subsample of study participants. Bar plots showing the sample distribution by pig age and colored by study. (A) distribution of all samples included, (B) distribution of all control samples, (C) distribution of control samples after subsampling for days with samples size over 50.

**FIG S2** Dynamic development of the microbial alpha diversity in the swine gut. Box plots showing the Pielou’s evenness (A, assessment of evenness), the number of observed features (B, quantitative assessment of richness), and Shannon diversity (C, quantitative assessment of richness and evenness combined) in the swine gut microbiota from birth to marketing. Spearman’s rho show the positive correlations between alpha diversity indices and pig age.

**FIG S3** Dynamic effects of fecal microbiota transplantation (FMT) on microbial alpha diversity and microbiota age. Box plots show the effects of FMT on Faith phylogenetic diversity (A), Pielou’s evenness (B), the number of observed features (C), and Shannon diversity (D) in the pig gut microbiota over time. (E) Linear models show the effects of antibiotics on microbiota age of pigs. Control samples and FMT-treated samples were from the same study.

**FIG S4** Dynamic effects of diet restriction on alpha diversity of the fecal microbiota by pig age. Box plots show the effects of diet restriction on Faith’s phylogenetic diversity (A), Pielou’s evenness (B), the number of observed OTUs (C), and the Shannon diversity index (D) in the pig gut microbiota over time. Control samples and diet restriction samples were from the same study.

**FIG S5** Temporal development of the swine gut microbiota. (A) Bar plots of the relative abundance of the 25 most relatively abundant genera over the course of time. Each bar represent the average relative abundance of each genus in the fecal microbiotas of control samples from untreated pigs at each time point. (B) Beta regression coefficients for the 62 most relatively abundant genera. Values > 0 indicate genera that increase in relative abundance over time and values < 0 are those that decrease as the pigs age.

**FIG S6** The gut microbiota beta diversity stabilizes after 40-50 days after birth. (A-D) Principal coordinates analysis (PCoA) of beta dissimilarities between control samples colored by growth stages (A, B) and ages (C, D). (E, F) Heatmaps depicting the mean pairwise z-score for similarity between control samples from each time point, calculated as (1 - beta dissimilarity). Jaccard dissimilarity was used in panels (A, C, E). Weighted UniFrac distance was used in panels (B, D, F). All statistical comparisons are presented in TABLE S3.

**FIG S7** PERMANOVA for the effect of study and 16S rRNA gene hypervariable region sequenced on the swine gut microbiota at weaning day. Principal coordinates analysis (PCoA) plots showing the dissimilarities or distances between control samples from untreated pigs colored by study (A, C, E, G) and hypervariable region sequenced (B, D, F, H). Bray-Curtis dissimilarity was used for panels (A, B). Jaccard dissimilarity index was used for panels (C, D). Weighted UniFrac distance was used for panels (E, F). Unweighted UniFrac distance was used for panels (G, H).

**FIG S8** The gut microbial community development through stage-specific DMM clusters. (A) Distribution of control samples in the identified 10 Dirichlet Multinomial Mixtures (DMM) clusters (y axis) using the DMM model at each time point (x axis). Sizes of circles represent number of samples of each cluster at each time point. All of the samples in this panel were from the three largest studies, each of which included samples from all four phases of growth. (B-D) Distribution of control fecal samples from untreated pigs for adjacent growth phases in the DMM clusters (y axis) at each time point (x axis). Suckling and weaning for panel (B), weaning and growing for panel (C), growing and finishing for panel (D). (E) Distribution of fecal samples in the DMM clusters (x axis) from different treatments (y axis). (F-H) Box plots of alpha diversity, measured by the Shannon diversity index (E), number of observed OTUs (F), and Pielou’s evenness (G), between each DMM clusters. Boxes show median and interquartile ranges (IQR), whiskers show ± 1.5 IQR from the quartiles. Statistics in TABLE S8.

**FIG S9** The random forest models are more robust in predicting early fecal samples than later ones. Microbiota age predictions for samples in the external validation dataset separated by early samples (A, age < 80 days) and late samples (B, age > 80 days).

**FIG S10** The superiority of the RF models created with all time-points over models created with sparse time points or the single largest data set. Microbiota age for samples in the external validation dataset predicted using the random forest models created with the single largest data set (A, Holman, et al., mSystems, 2021), or a data set with 10 randomly selected time points (B, days 7, 21, 35, 49, 61, 84, 99, 112, 130, 146).

**FIG S11** The dynamic “core” microbiota. The Venn diagram and UpSet plot shows the intersection and concatenation of the “core” microbiota from different growth stages. The “core” microbiota is defined as taxa present in at least 90% of samples within each stage [Created using ehbio.com/ImageGP].
