# Supplementary material for: Meta-analysis reveals the predictable dynamic development of the gut microbiota in commercial pigs
Source: Microbiol Spectr. 2023 Oct 10;11(6):e01722-23. doi: 10.1128/spectrum.01722-23 (PMC10715009; doi:10.1128/spectrum.01722-23)
Supplement: Supplemental figures — Fig. S1 to S11. [file spectrum.01722-23-s0002.pdf]

FIG S1

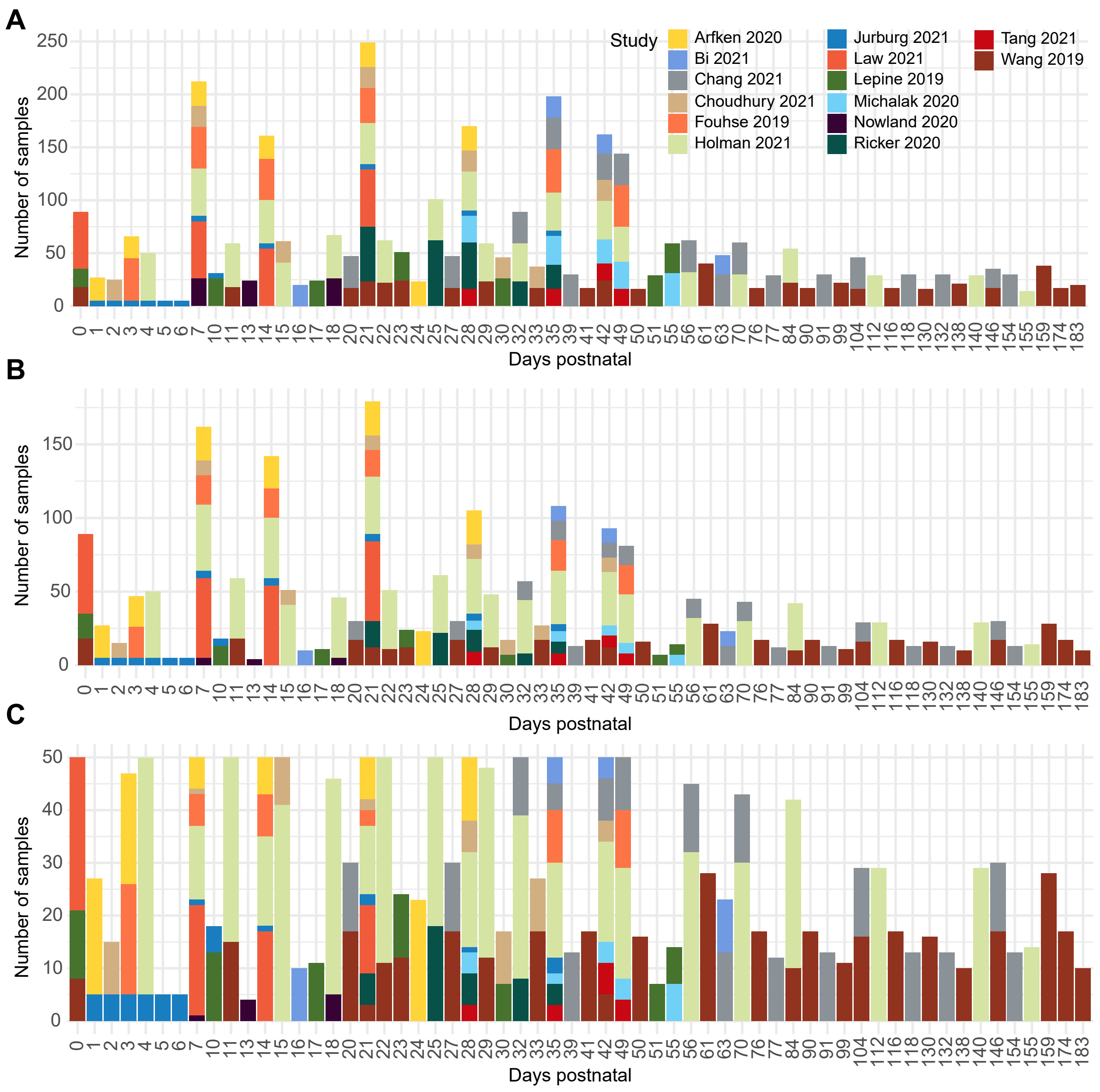
A
B
C
Number of samples
Days postnatal
Study
Arfken 2020
Bi 2021
Chang 2021
Choudhury 2021
Fouhse 2019
Holman 2021
Jurburg 2021
Law 2021
Lepine 2019
Michalak 2020
Nowland 2020
Ricker 2020
Tang 2021
Wang 2019

FIG S2

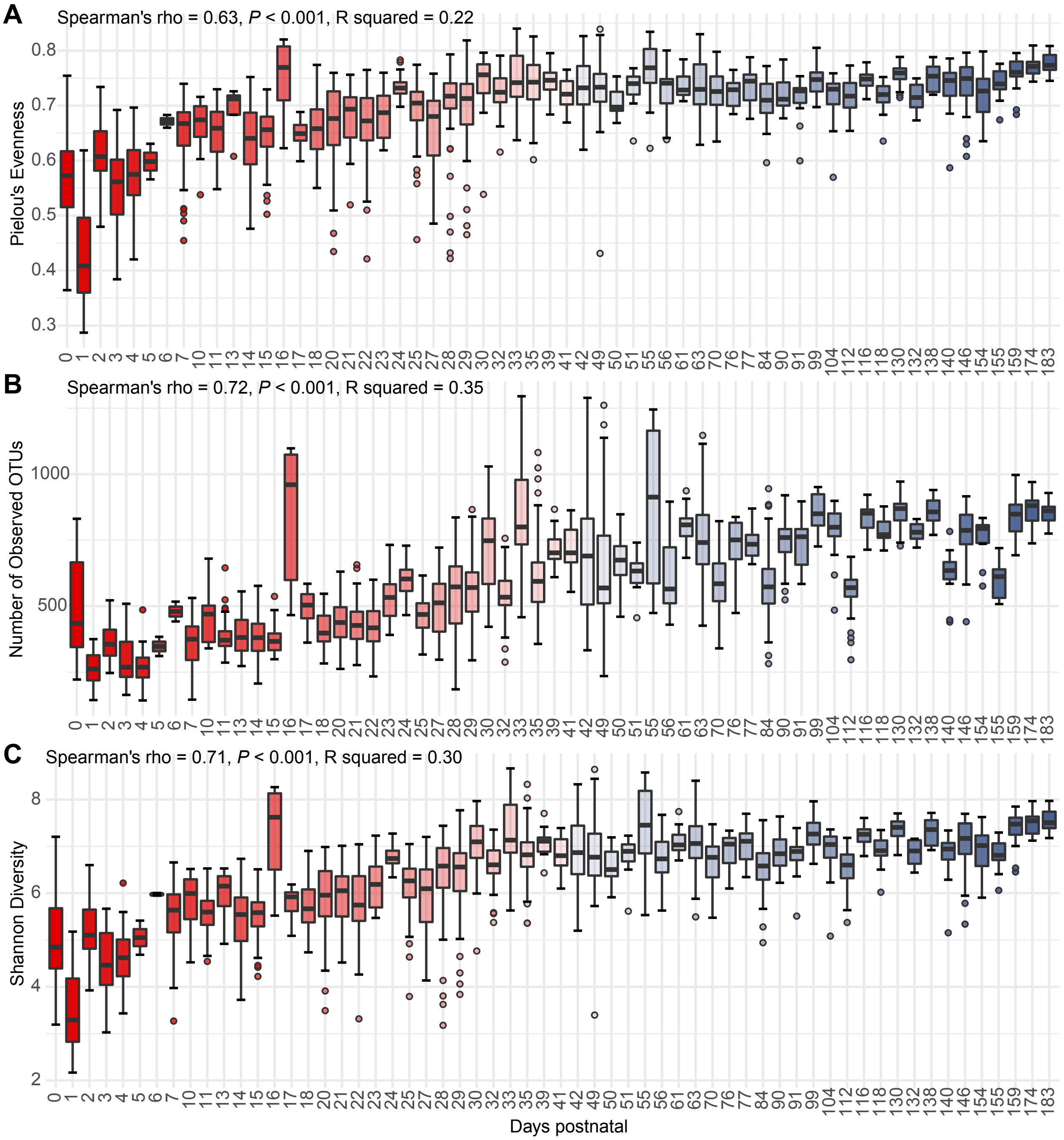

A
Spearman's rho = 0.63, P < 0.001, R squared = 0.22
Pielou's Evenness
B
Spearman's rho = 0.72, P < 0.001, R squared = 0.35
Number of Observed OTUs
C
Spearman's rho = 0.71, P < 0.001, R squared = 0.30
Shannon Diversity
Days postnatal

FIG S3

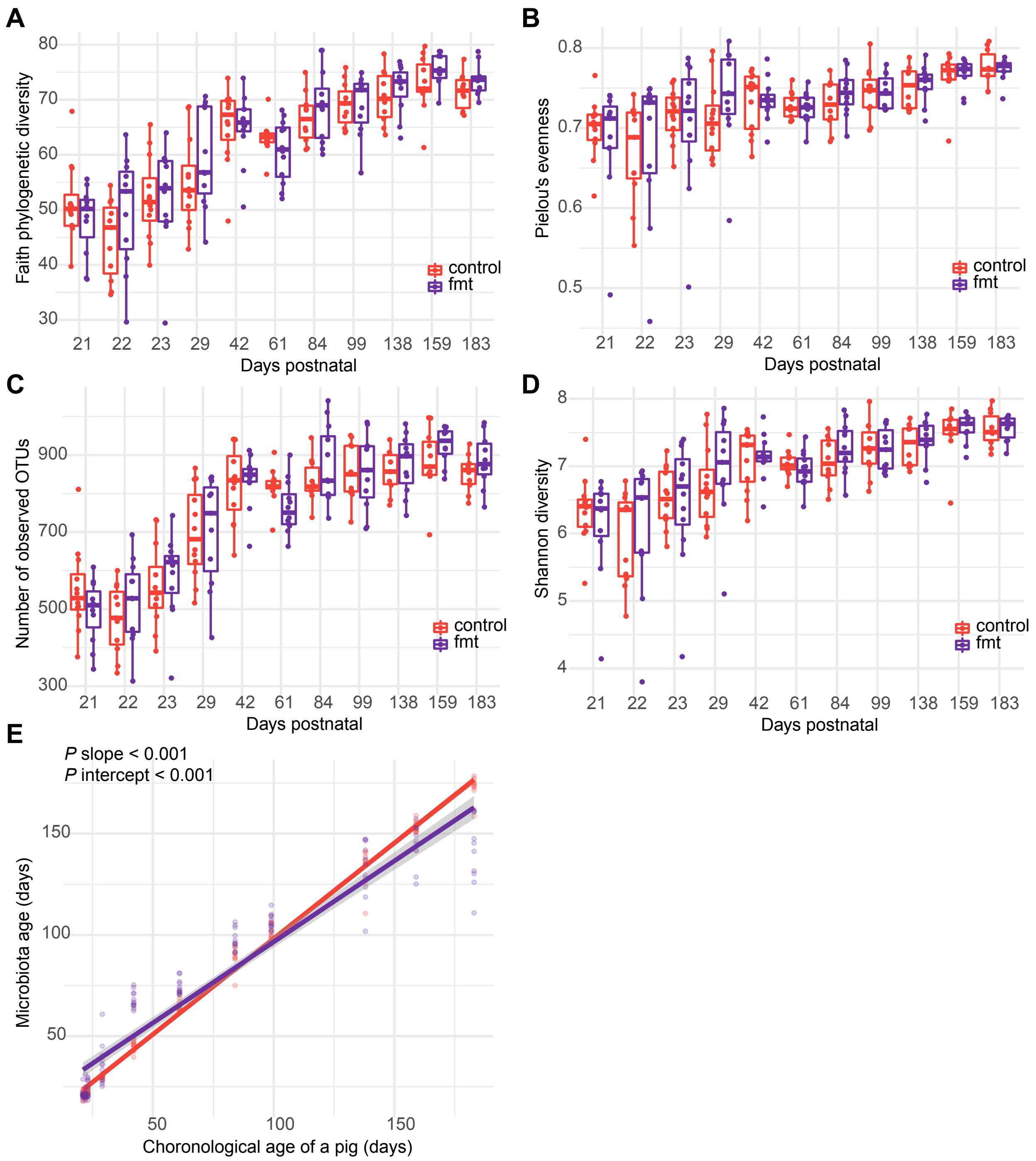
A
Faith phylogenetic diversity
Days postnatal
control
fmt
B
Pielou's evenness
Days postnatal
control
fmt
C
Number of observed OTUs
Days postnatal
control
fmt
D
Shannon diversity
Days postnatal
control
fmt
E
P slope < 0.001
P intercept < 0.001
Microbiota age (days)
Choronological age of a pig (days)

FIG S4

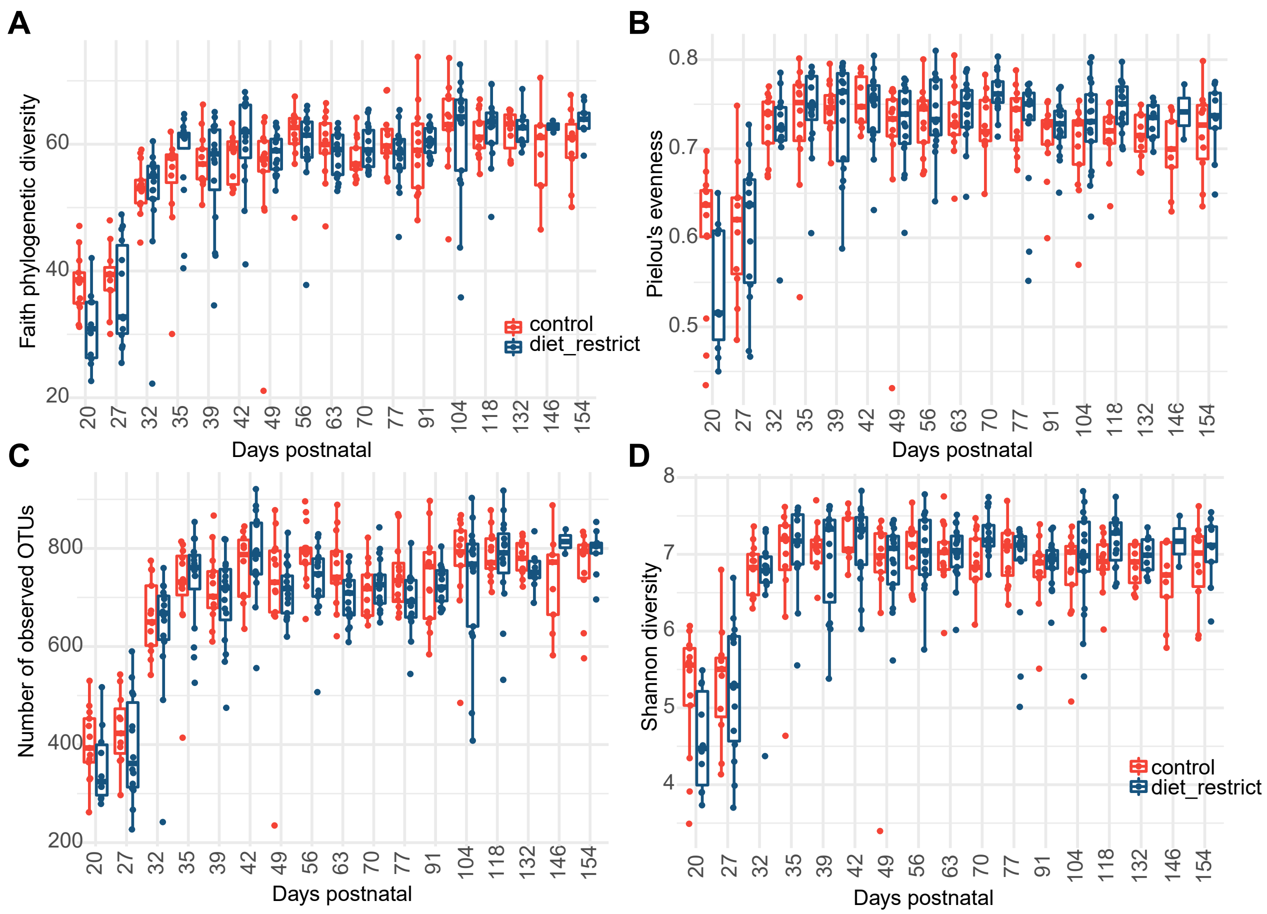
A
Faith phylogenetic diversity
20
40
60
control
diet_restrict
20 27 32 35 39 42 49 56 63 70 77 91 104 118 132 146 154
Days postnatal
B
Pielou's evenness
0.5
0.6
0.7
0.8
20 27 32 35 39 42 49 56 63 70 77 91 104 118 132 146 154
Days postnatal
C
Number of observed OTUs
200
400
600
800
20 27 32 35 39 42 49 56 63 70 77 91 104 118 132 146 154
Days postnatal
D
Shannon diversity
4
5
6
7
8
control
diet_restrict
20 27 32 35 39 42 49 56 63 70 77 91 104 118 132 146 154
Days postnatal

FIG S5

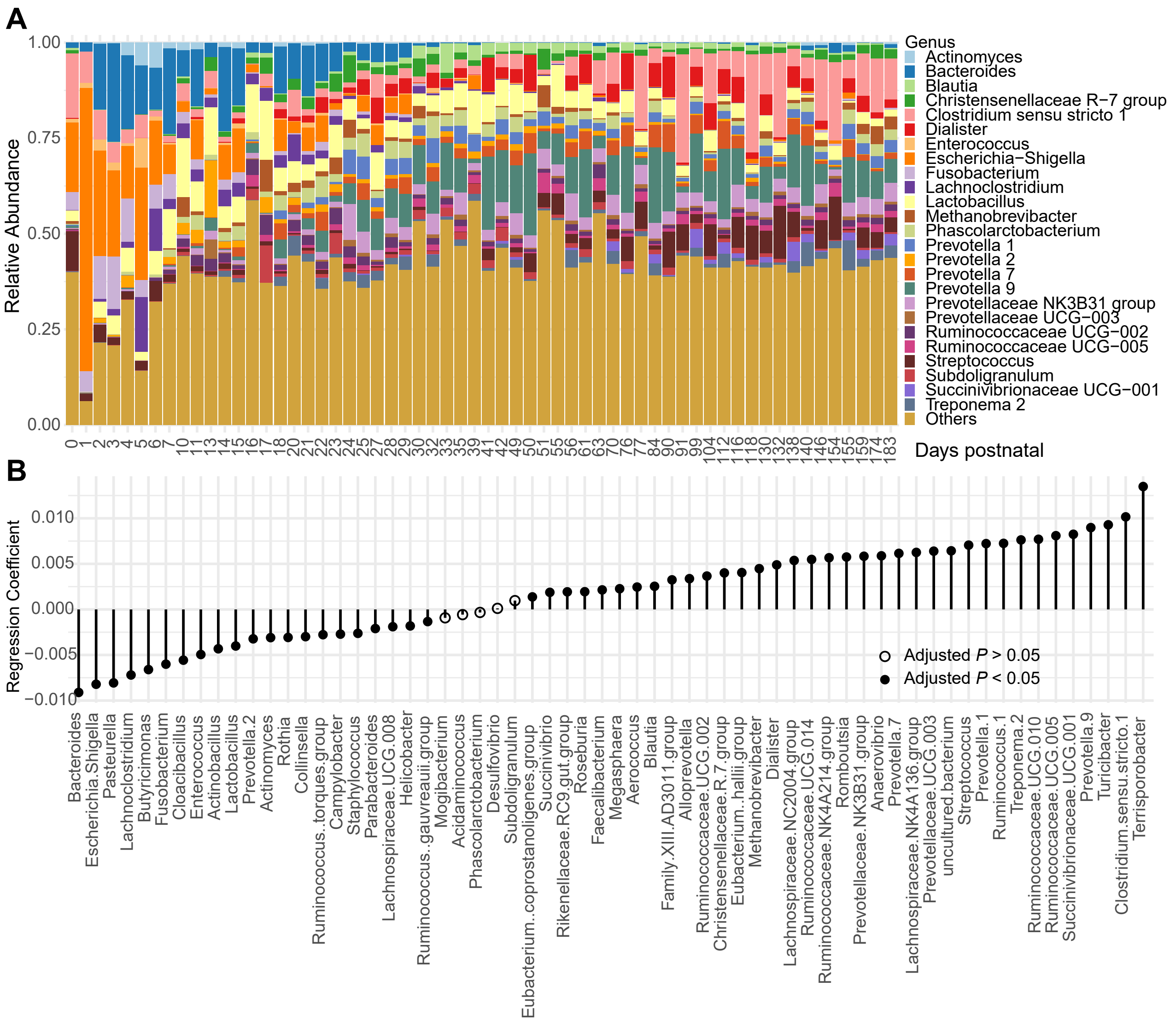

A
Relative Abundance
Days postnatal
Genus
Actinomyces
Bacteroides
Blautia
Christensenellaceae R−7 group
Clostridium sensu stricto 1
Dialister
Enterococcus
Escherichia−Shigella
Fusobacterium
Lachnoclostridium
Lactobacillus
Methanobrevibacter
Phascolarctobacterium
Prevotella 1
Prevotella 2
Prevotella 7
Prevotella 9
Prevotellaceae NK3B31 group
Prevotellaceae UCG−003
Ruminococcaceae UCG−002
Ruminococcaceae UCG−005
Streptococcus
Subdoligranulum
Succinivibrionaceae UCG−001
Treponema 2
Others
B
Regression Coefficient
Adjusted P > 0.05
Adjusted P < 0.05
Bacteroides
Escherichia.Shigella
Pasteurella
Lachnoclostridium
Butyricimonas
Fusobacterium
Cloacibacillus
Enterococcus
Actinobacillus
Lactobacillus
Prevotella.2
Actinomyces
Rothia
Collinsella
Ruminococcus..torques.group
Campylobacter
Staphylococcus
Parabacteroides
Lachnospiraceae.UCG.008
Helicobacter
Ruminococcus..gauvreauii.group
Mogibacterium
Acidaminococcus
Phascolarctobacterium
Desulfovibrio
Subdoligranulum
Eubacterium..coprostanoligenes.group
Succinivibrio
Rikenellaceae.RC9.gut.group
Roseburia
Faecalibacterium
Megasphaera
Aerococcus
Blautia
Family.XIII.AD3011.group
Alloprevotella
Ruminococcaceae.UCG.002
Christensenellaceae.R.7.group
Eubacterium..hallii.group
Methanobrevibacter
Dialister
Lachnospiraceae.NC2004.group
Ruminococcaceae.UCG.014
Ruminococcaceae.NK4A214.group
Romboutsia
Prevotellaceae.NK3B31.group
Anaerovibrio
Prevotella.7
Lachnospiraceae.NK4A136.group
Prevotellaceae.UCG.003
uncultured.bacterium
Streptococcus
Prevotella.1
Ruminococcus.1
Treponema.2
Ruminococcaceae.UCG.010
Ruminococcaceae.UCG.005
Succinivibrionaceae.UCG.001
Prevotella.9
Turicibacter
Clostridium.sensu.stricto.1
Terrisporobacter

FIG S6

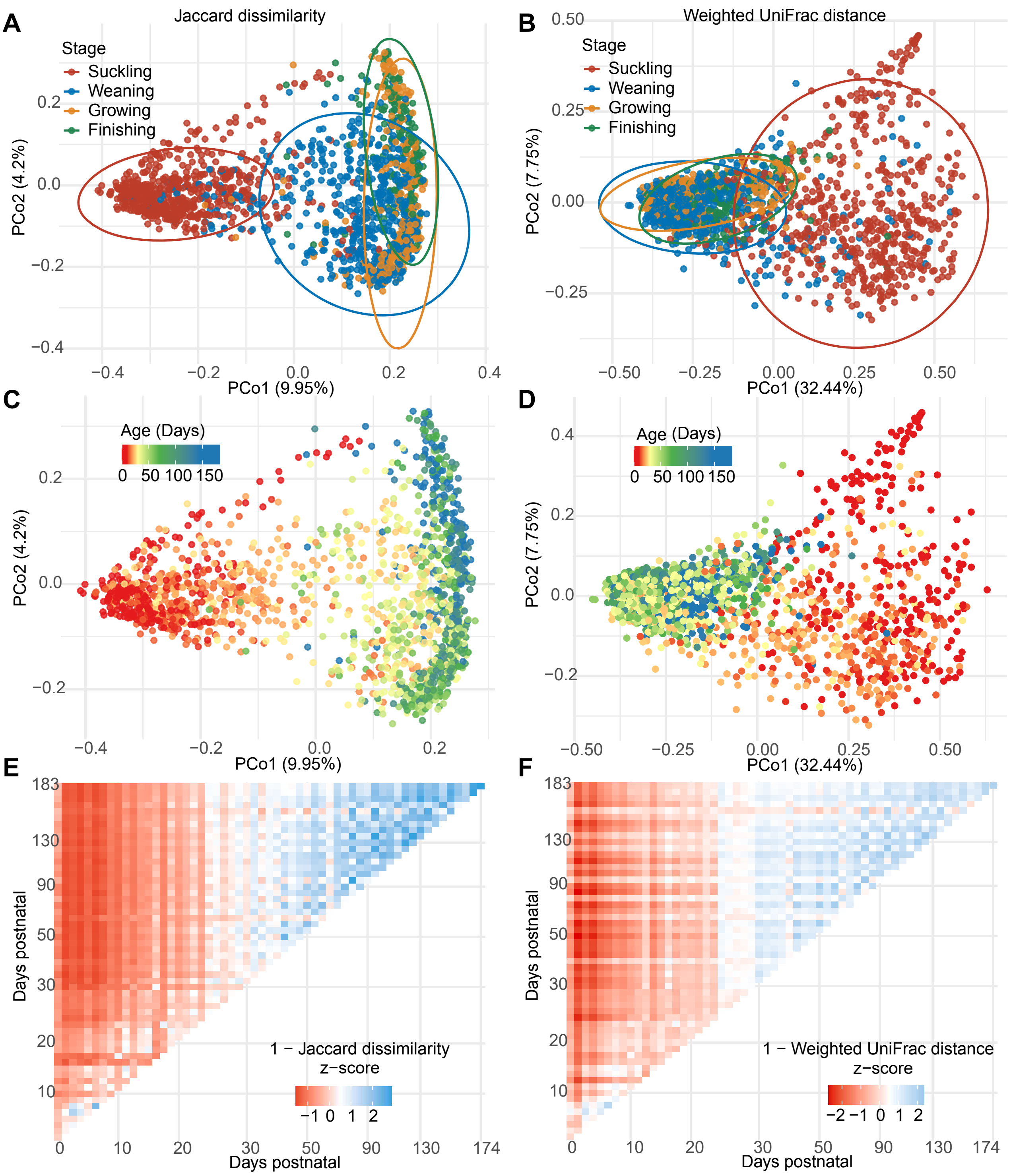
A
Jaccard dissimilarity
Stage
Suckling
Weaning
Growing
Finishing
PCo2 (4.2%)
PCo1 (9.95%)
B
Weighted UniFrac distance
PCo2 (7.75%)
PCo1 (32.44%)
C
Age (Days)
0 50 100 150
D
E
Days postnatal
1 − Jaccard dissimilarity
z−score
F
1 − Weighted UniFrac distance
z−score

FIG S7

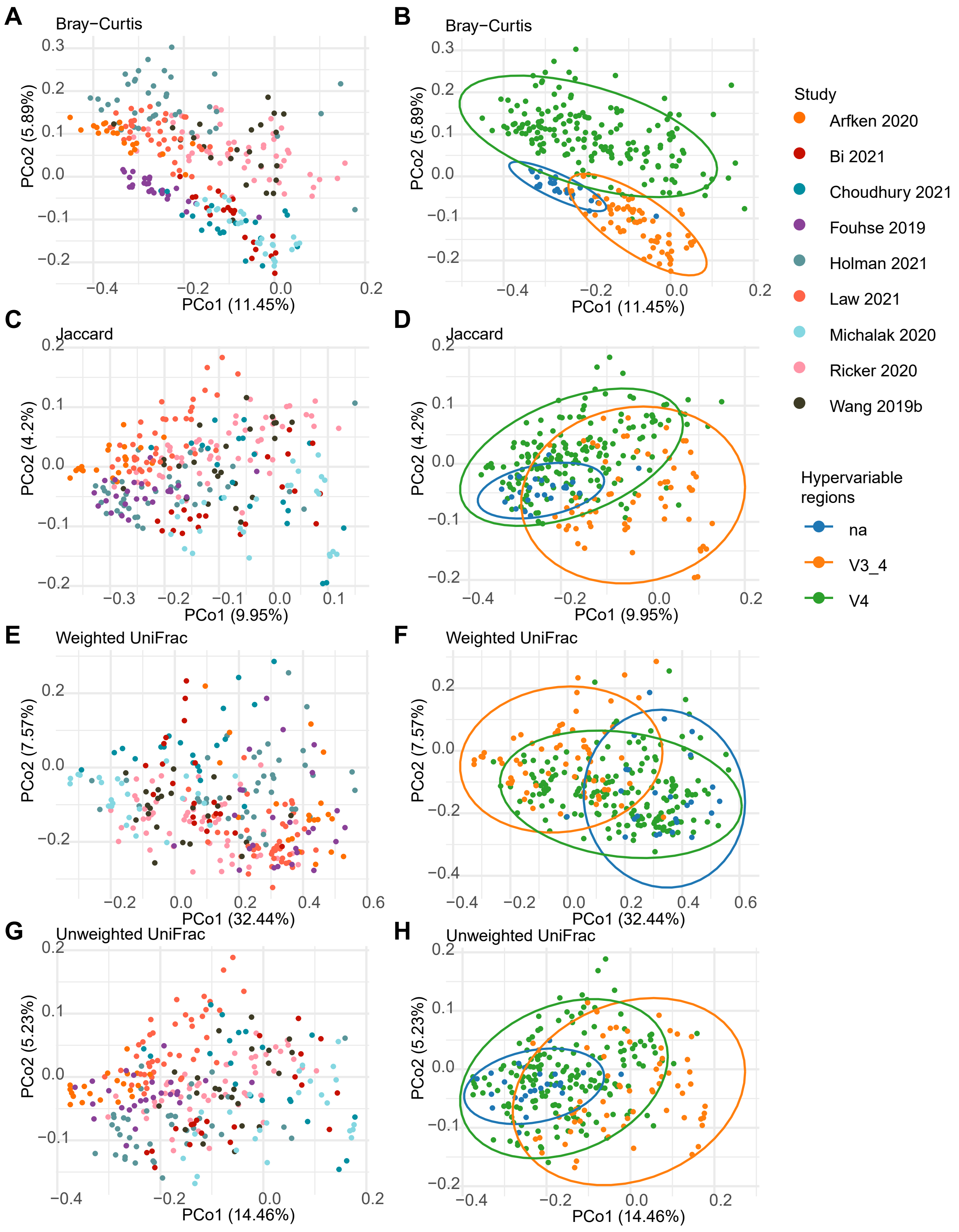
A
Bray−Curtis
B
Bray−Curtis
PCo1 (11.45%)
PCo2 (5.89%)
C
Jaccard
D
Jaccard
PCo1 (9.95%)
PCo2 (4.2%)
E
Weighted UniFrac
F
Weighted UniFrac
PCo1 (32.44%)
PCo2 (7.57%)
G
Unweighted UniFrac
H
Unweighted UniFrac
PCo1 (14.46%)
PCo2 (5.23%)
Study
Arfken 2020
Bi 2021
Choudhury 2021
Fouhse 2019
Holman 2021
Law 2021
Michalak 2020
Ricker 2020
Wang 2019b
Hypervariable regions
na
V3_4
V4

FIG S8

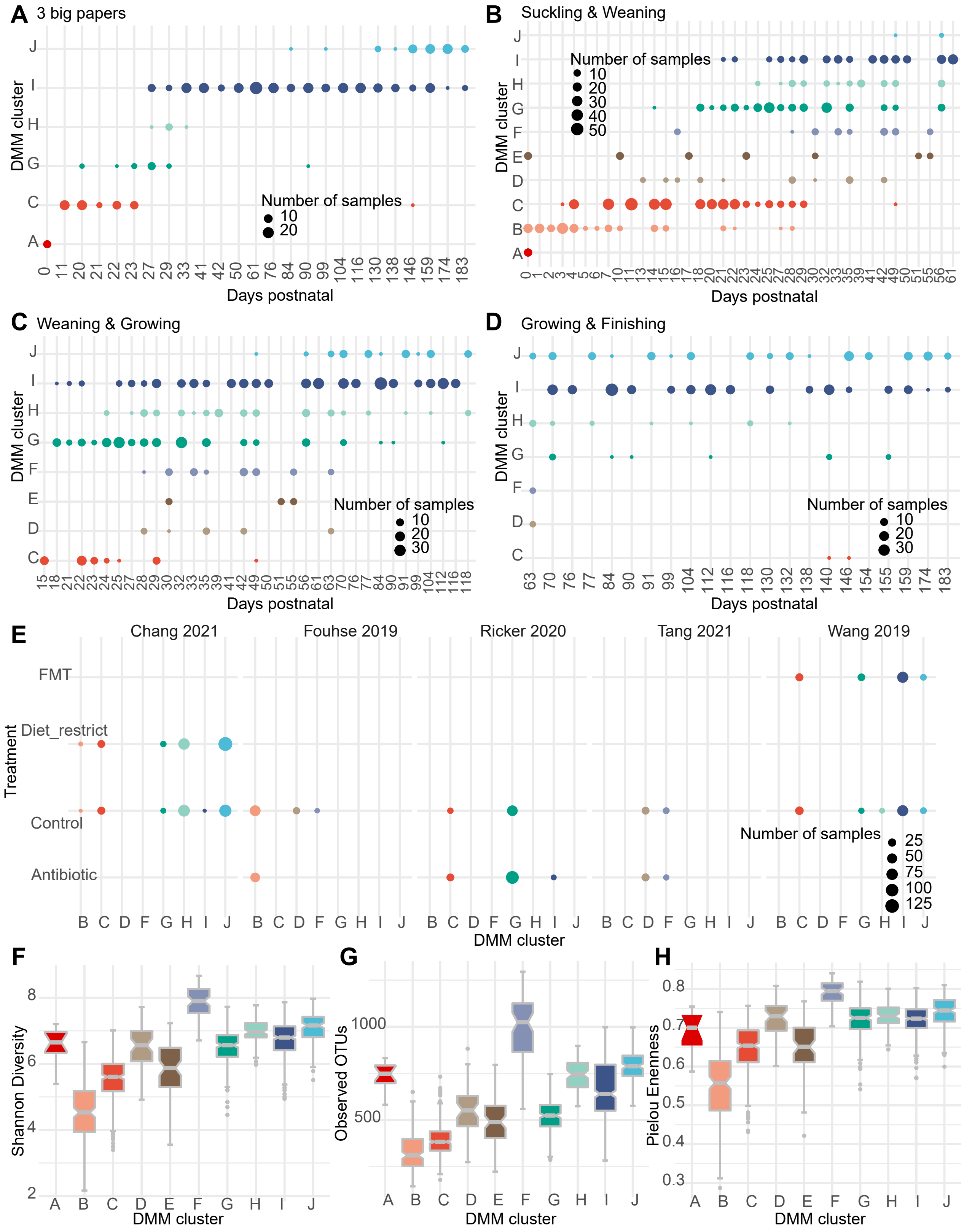
A 3 big papers
B Suckling & Weaning
C Weaning & Growing
D Growing & Finishing
E
F
G
H
DMM cluster
Days postnatal
Number of samples
Chang 2021
Fouhse 2019
Ricker 2020
Tang 2021
Wang 2019
FMT
Diet_restrict
Control
Antibiotic
Treatment
Shannon Diversity
Observed OTUs
Pielou Enenness

FIG S9

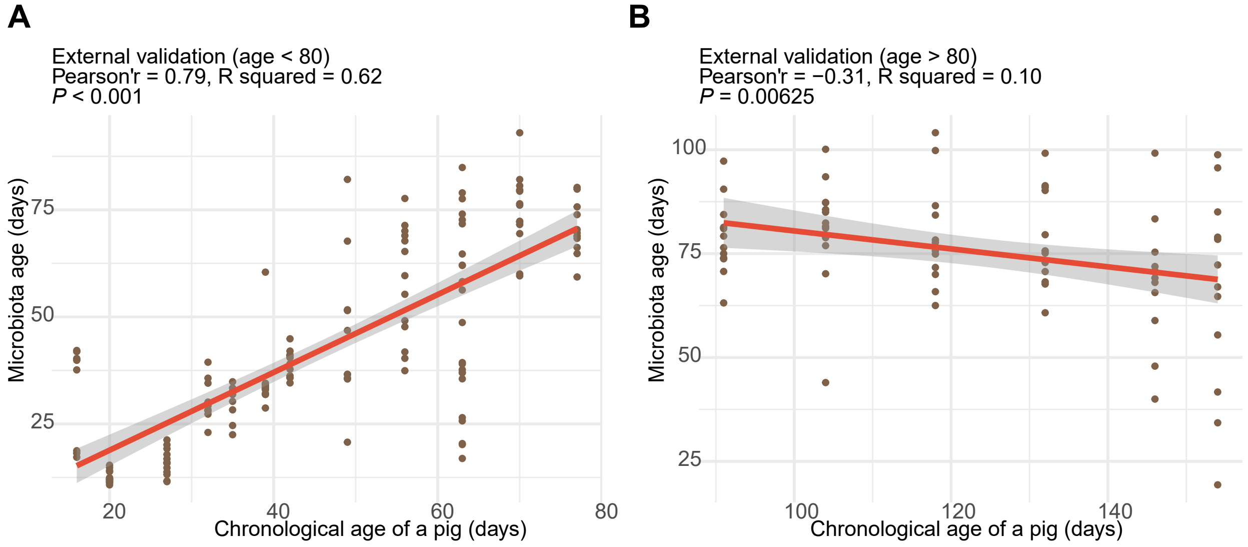
A
External validation (age < 80)
Pearson'r = 0.79, R squared = 0.62
P < 0.001
Microbiota age (days)
Chronological age of a pig (days)
25
50
75
20
40
60
80
B
External validation (age > 80)
Pearson'r = −0.31, R squared = 0.10
P = 0.00625
Microbiota age (days)
Chronological age of a pig (days)
25
50
75
100
100
120
140

FIG S10

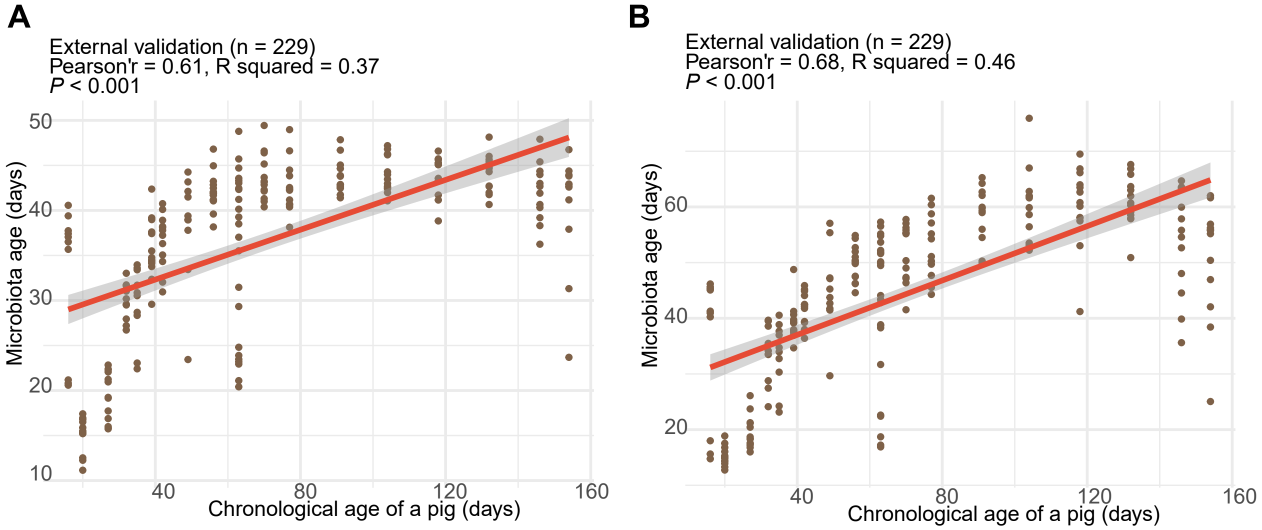
A
External validation (n = 229)
Pearson'r = 0.61, R squared = 0.37
P < 0.001
Microbiota age (days)
50
40
30
20
10
40
80
120
160
Chronological age of a pig (days)
B
External validation (n = 229)
Pearson'r = 0.68, R squared = 0.46
P < 0.001
Microbiota age (days)
60
40
20
40
80
120
160
Chronological age of a pig (days)

FIG S11

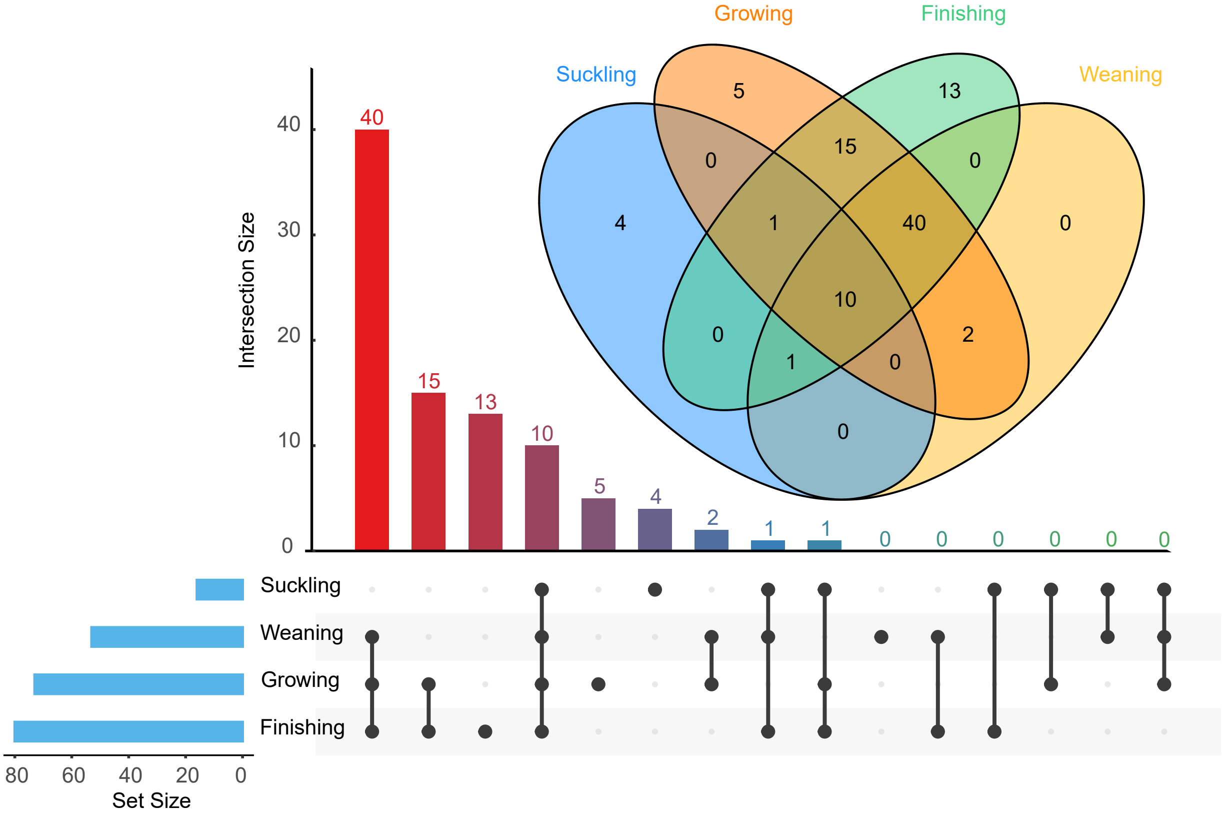

Suckling
Growing
Finishing
Weaning
5
13
0
15
0
4
1
40
0
10
0
2
1
0
0
Intersection Size
40
30
20
10
0
40
15
13
10
5
4
2
1
1
0
0
0
0
0
0
Suckling
Weaning
Growing
Finishing
80
60
40
20
0
Set Size
